# The Perceptions of Foreign‐Born Arab Americans on Dementia and Mental Health Research: A Focus Group Study

**DOI:** 10.1002/alz70858_107203

**Published:** 2025-12-26

**Authors:** Sumiyyah M Zimami, Hala J Darwish, Kristine J Ajrouch, Sarah Abboud, Daniel Velez Ortiz

**Affiliations:** ^1^ University of Michigan, Ann Arbor, MI, USA; ^2^ Jazan University, Jazan, Southwestern, Saudi Arabia; ^3^ Eastern Michigan University, Ypsilanti, MI, USA; ^4^ University of Illinois Chicago, Chicago, IL, USA; ^5^ Michigan State University, East Lansing, MI, USA

## Abstract

**Background:**

Arab Americans represent a rapidly growing subpopulation in the United States, with an estimated 3 million individuals. However, they face unique challenges in understanding and engaging in dementia and mental health research. Despite increasing recognition of these issues, there remains a scarcity of research focused on dementia and mental health within this community. Many Arab Americans hesitate to participate in research in general and in dementia and mental health research in particular, which hinders the efforts to understand and address their healthcare needs. Therefore, the aim of this study is to explore the perceptions and knowledge of foreign‐born Arab Americans about research on dementia and mental health.

**Methods:**

Three in‐person focus group discussions (*n* = 12, *n* = 10, and *n* = 6 participants) were conducted in Arabic and English, guided by semi‐structured interviews. A total of 28 participants (18 women) aged 30 years and older took part. The discussions were audio‐recorded, transcribed, translated, and analyzed using Atlas.ti software. Inductive thematic analysis identified the commonly recurring themes. A concluding formal educational session was held to raise participants’ awareness about the topic and answer their questions.

**Results:**

A preliminary analysis of the focus groups revealed five major themes: a varied understanding of research and its purpose, a lack of awareness about early dementia symptoms, prevalent fears and stigma surrounding dementia and mental health, barriers to research participation, including lack of knowledge and apprehension, mistrust, and competing life priorities, and strategies to enhance participation, including community education, increased outreach programs, and incentives.

**Conclusions:**

Cultural factors play a critical role in Arabs’ perceptions of research, dementia, and mental health. Participants valued the concluding educational session, which provided an opportunity to ask questions and engage in discussions. Addressing stigma through culturally tailored education, promoting research literacy, and increasing community engagement efforts are essential strategies for improving participation in dementia and mental health research. Future initiatives should prioritize trust‐building, community‐led interventions, and multilingual resources to ensure accessibility and inclusivity in healthcare research.

